# Oral microbiota and urinary system diseases: from mechanistic insights to clinical implications—a comprehensive review

**DOI:** 10.3389/fdmed.2026.1803961

**Published:** 2026-04-22

**Authors:** Haoling Chen, Yanan Chen, Yiwen Liu, Chuxiao Chen

**Affiliations:** 1 Department of Pediatric Dentistry, Stomatological Hospital, School of Stomatology, Southern Medical University, Guangzhou, Guangdong, China; 2 Department of Endodontics, Stomatological Hospital, School of Stomatology, Southern Medical University, Guangzhou, Guangdong, China; 3 Organ Transplant Center, The First Affiliated Hospital, Sun Yat-sen University, Guangzhou, Guangdong, China

**Keywords:** benign prostate diseases, chronic kidney disease, mechanistic pathways, oral microbiome, oral-urinary axis, pediatric Henoch-Schönlein purpura nephritis, urolithiasis, urologic cancers

## Abstract

The human oral microbiome has attracted considerable attention due to its role in oral health and potential implications for systemic diseases. Oral microbes provide real-time insights into health and disease status, making them valuable for early disease risk stratification and treatment outcome prediction. Accumulating evidence indicates that oral microbiota contribute to the pathogenesis of urinary system diseases. Notably, in pediatric populations, the oral microbiome—shaped by age, feeding patterns, and immune maturation—may modulate susceptibility to renal-related systemic conditions; clinical observations specifically link untreated early childhood caries to an increased risk of Henoch-Schönlein purpura nephritis (HSPN). This review critically appraises the existing literature to clarify the nature and magnitude of the association between the oral microbiome and urinary system diseases, including chronic kidney disease, urolithiasis, benign prostatic hyperplasia, and urologic cancers, as well as pediatric HSPN. We also analyze the potential mechanisms through which the oral microbiota are involved in the pathogenesis and progression of these relevant diseases, and explore its potential implications for the prevention, diagnosis, and management of urinary system disorders.

## 1 Introduction

The human microbiota consist of all the microbes within the body, including bacteria, fungi, protozoa, and viruses, which exert homeostatic effects on the healthy immune system (1). The oral cavity harbors a substantial abundance of microorganisms, owing to its specific anatomical structures, connection to the external environment, and humid conditions, which constitute one of the five research priorities of the Human Microbiome Project (2, 3). The synergy and interaction of various oral microorganisms contribute to the resistance of human body to the undesirable stimulation outside. Nevertheless, the imbalance of oral microbial flora leads to oral diseases and systemic diseases (4). The theory of oral focal infection was proposed in the early 1890s, suggesting that dental plaque and its metabolites enter the bloodstream and induce various systemic diseases, including nephritis, rheumatoid arthritis, and endocarditis (5). As oral microbes offer real-time indications of human health and disease conditions, they hold significant value in the early warning of disease risks and the prediction of therapeutic outcomes (4, 6, 7). These insights underscore the paramount significance of maintaining microbial homeostasis within the oral cavity as a measure to reinforce overall health and hinder the onset of a diverse array of diseases.

The urinary system is a key organ system responsible for regulating water-salt metabolism and acid-base balance via excretion, and its dysfunction is associated with high morbidity and mortality worldwide (8). Mounting clinical and epidemiological evidence has linked poor oral health (e.g., dental caries, periodontitis) to multiple urinary system diseases, and the proposed “oral-urinar*y* axis” has become a research hotspot in oral-systemic microbial interactions (9). Dysbiosis of the oral microbiota may contribute to an increased risk of chronic kidney disease (CKD), urolithiasis, benign prostate diseases, urologic cancers, and pediatric Henoch-Schönlein purpura nephritis (HSPN) (9–11). Notably, the oral microbiome in children is dynamically regulated by age, feeding patterns and immune maturation, and its dysbiosis is a potential modifiable risk factor for pediatric HSPN. This review is based on the core framework of the oral-urinar*y* axis, systematically summarizes the epidemiological and clinical evidence of the association between oral microbiota and major urinary system diseases, critically evaluates the methodological quality and evidence strength of existing studies, elaborates on the disease-specific pathogenic mechanisms, and finally discusses the challenges and translational prospects of oral microbiome-targeted interventions for urinary system diseases. To improve clarity, facilitate cross-study comparison, and strengthen the overall evidence synthesis, the key studies investigating the association between oral microbiota and urinary system diseases are summarized in Table 1.

**Table 1:** Key studies investigating the association between oral microbiota and urinary system diseases.

Disease type	Study design	Oral sampling site	Microbiome/ measurement method	Principal findings	Reference
CKD	Cross-sectional	Saliva	16S rRNA gene sequencing	Increased oral microbial diversity in CKD; enriched genera: *Streptococcus, Leptotrichia, Actinomyces*; reduced: *Haemophilus, Prevotella*.	(69, 70)
CKD & IgAN	Cross-sectional	Oral cavity, tonsils	NR	Higher prevalence of periodontal pathogens (Porphyromonas gingivalis, Tannerella forsythia, Treponema denticola, Candida albicans) in CKD; cnm-positive Streptococcus mutans is associated with IgAN.	(32–34)
IgAN	Cross-sectional	Oral cavity	16S rRNA gene sequencing	Enriched: *Capnocytophaga*; depleted: Rothia, Dialister, Actinomyces.	(36)
Urolithiasis	Cross-sectional	Saliva	16S rRNA gene sequencing	Higher abundance of *Porphyromonas, Neisseria*; lower *Corynebacterium*, *Leptotrichia, Prevotella*; reduced microbial richness in uric acid stones.	(40)
Benign prostatic diseases	Cross-sectional observational	Subgingival plaque, prostatic secretion	Microbiological identification	70.8% of prostatic secretion samples positive for oral pathogens (*Porphyromonas gingivalis, Prevotella intermedia, Treponema denticola*); *Fusobacterium nucleatum* detected in inflamed prostate tissue.	(45)
HSPN	Retrospective observational	NR	Serological testing	18.9% of patients had streptococcal infection, which is linked to HSP/HSPN onset and recurrence.	(62)
HSPN	Case-series observational	Skin and renal biopsies	Immunohistochemistry	IgA-bound streptococcal M protein present in 80% of skin lesions and 54% of renal lesions.	(64)
HSPN	Cross-sectional	Oral swabs, tongue coating	16S rRNA gene sequencing	High prevalence of caries and apical periodontitis; increased oral microbial diversity; dominant phyla: *Firmicutes, Proteobacteria, Bacteroidetes.*	(21)

CKD, chronic kidney disease; eGFR, estimated glomerular filtration rate; IgAN, IgA nephropathy; NR, not reported; HSPN, Henoch-Schönlein purpura nephritis.

## 2 The oral-urinary microbiome axis: a conceptual framework

**Figure 1:**
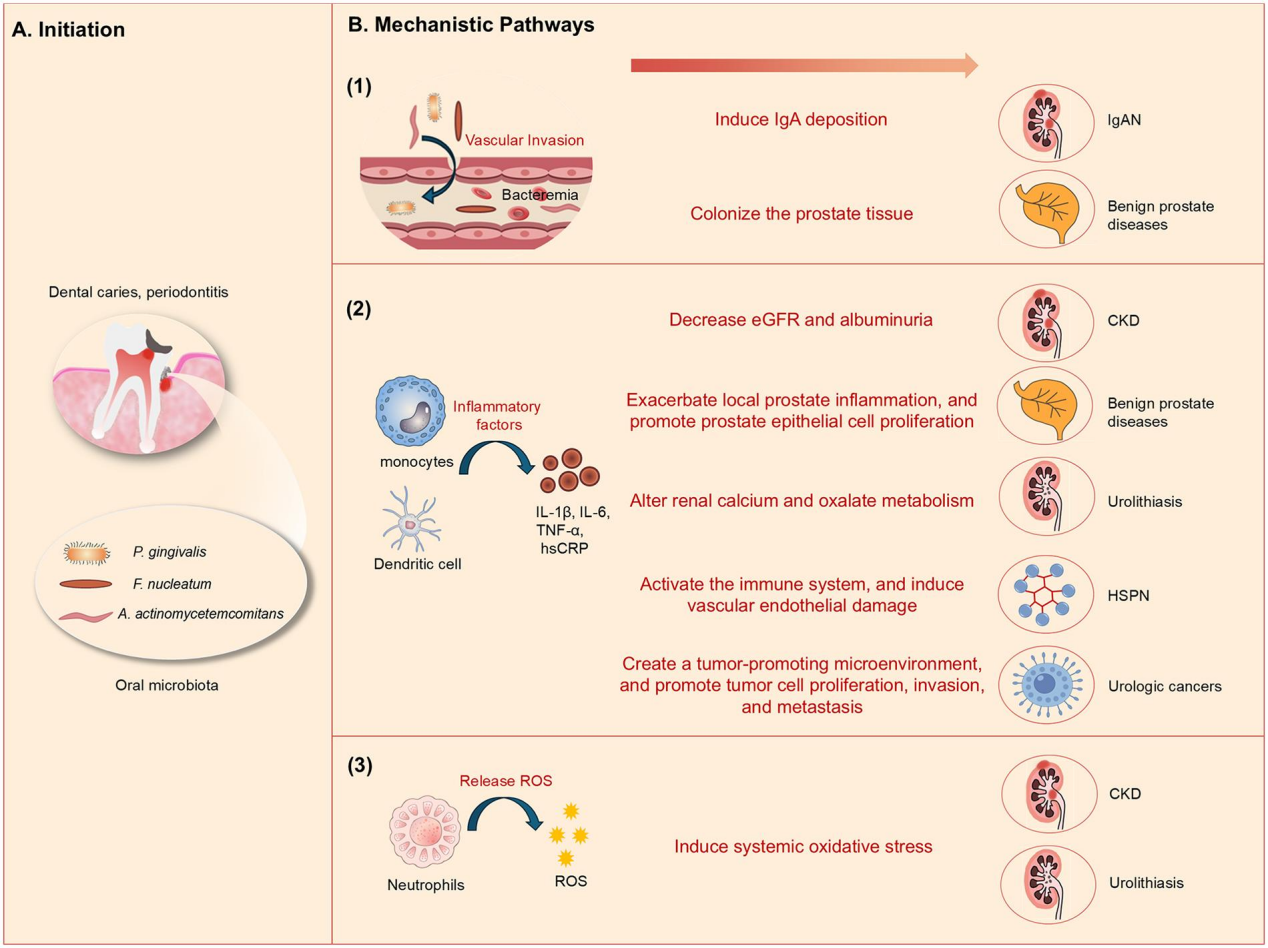
The oral-urinary microbiome axis: from pathogenic initiation to disease manifestation. **(A)** Initiation of oral dysbiosis. Dental caries and periodontitis disrupt the oral mucosal barrier, leading to the outgrowth of key pathogenic taxa including *Porphyromonas gingivalis*, *Fusobacterium nucleatum*, and *Aggregatibacter actinomycetemcomitans*. These dysbiotic microbiota breach the barrier, entering the systemic circulation via vascular invasion and bacteremia. **(B)** Core mechanistic pathways mediating the oral-urinary axis. Three primary cascades drive pathological progression: **(1)** Bacteria invasion: oral bacteria translocate into the bloodstream, colonize distal tissues, and lead to distinct urinary tract diseases: vascular invasion mediates IgA deposition in IgAN and tissue colonization in benign prostate diseases; **(2)** Inflammatory response: monocytes and dendritic cells release pro-inflammatory cytokines (IL-1β, IL-6, TNF-α, hsCRP) that drive decreased eGFR and albuminuria in CKD, exacerbates prostate inflammation in benign prostate diseases, alters renal calcium/oxalate metabolism in urolithiasis, activates immune-mediated vascular damage in HSPN, and creates a tumor-promoting microenvironment in urologic cancers; **(3)** Oxidative stress response: neutrophils release ROS, inducing systemic oxidative stress contributing to CKD and urolithiasis. *P gingivalis, Porphyromonas gingivalis; F nucleatum, Fusobacterium nucleatum; A actinomycetemcomitans*, *Aggregatibacter actinomycetemcomitans*; CKD, chronic kidney disease; IgAN, IgA nephropathy; HSPN, Henoch-Schönlein purpura nephritis; ROS, reactive oxygen species; eGFR, estimated glomerular filtration rate; IL, interleukin; TNF-α, tumor necrosis factor-α; hsCRP, high-sensitivity C-reactive protein.

The oral-urinar*y* axis refers to the bidirectional regulatory network between the oral microbiome and the urinary system, which mediates the cross-talk between the two organ systems through bacterial dissemination, systemic inflammatory response, oxidative stress, and metabolic metabolite transmission (9). The pathogenic mechanisms of the oral-urinary microbiome axis are depicted in Figure 1. The oral cavity is an open microbial ecosystem, and its mucosal barrier is easily damaged by oral diseases (e.g., periodontitis, dental caries), leading to the entry of oral microbes and their metabolites into the systemic circulation via blood and lymphatic vessels (5, 12). These exogenous factors then reach the urinary system, where they induce local tissue damage, immune dysfunction, and metabolic disorders, ultimately promoting the initiation and progression of urinary system diseases (10, 11).

Conversely, the urinary system can also exert a retrograde regulatory effect on the oral microbiome: urinary system diseases often cause systemic metabolic disorders and immune suppression, which alter the oral microenvironment (e.g., saliva composition, pH value) and further exacerbate oral microbial dysbiosis, forming a vicious cycle (13). This conceptual framework provides a unified theoretical basis for interpreting the association between oral microbiota and various urinary system diseases, and all subsequent disease-specific discussions are grounded in this axis.

## 3 Oral microbiome: core features associated with systemic diseases

Since the 18th century, when bacteria in the oral cavity were first discovered, the oral microbiome has been a pivotal model for studying multispecies microbial communities (14, 15). It is a complex ecosystem composed of bacteria, fungi, viruses, protozoa, and archaea, which predominantly colonize the oral mucosa, dental plaque, and saliva (16, 17). Over 1,000 microbial species have been identified in the oral cavity via molecular analysis, and the core pathogenic taxa associated with systemic diseases include *Streptococcus mutans*, *Porphyromonas gingivalis*, *Aggregatibacter actinomycetemcomitans*, *Tannerella forsythia*, and *Fusobacterium nucleatum* (18–20).

Advances in next-generation sequencing (NGS) have expanded the understanding of oral microbial diversity, including the Candidate Phyla Radiation (CPR) and oral virome (21). However, only a small number of oral microbes have been confirmed to be associated with urinary system diseases, and their pathogenicity is closely related to their invasiveness, virulence factors, and ability to induce systemic inflammation (22).

The oral microbiome of healthy individuals is dominated by Actinobacteria, Firmicutes, Bacteroidetes, Proteobacteria, and Fusobacteria (23), and its homeostasis is regulated by endogenous (age, immune status) and exogenous (diet, antibiotic use, smoking) factors (4, 16). Dysbiosis of the oral microbiome—characterized by decreased microbial diversity, enrichment of pathogenic taxa, and depletion of beneficial taxa—is the core initiating factor for the activation of the oral-urinar*y* axis and the subsequent development of urinary system diseases (4). Oral sampling sites for microbiome analysis mainly include saliva, subgingival plaque, tongue coating, and oral swabs. Saliva and subgingival plaque are the most commonly used samples in studies on the oral-urinar*y* axis, as they can reflect the overall oral microbial profile and the abundance of periodontal pathogens, respectively (17).

## 4 Oral microbiome and urinary system diseases

### 4.1 Oral microbiome and CKD

CKD is defined as a glomerular filtration rate (GFR) < 60 mL/min/1.73 m^2^ or the presence of kidney damage markers (e.g., albuminuria) for ≥ 3 months, regardless of the underlying cause (24). It is a global public health concern with high progression risk to end-stage renal disease (ESRD, GFR < 15 mL/min/1.73 m^2^), which is associated with increased morbidity and mortality (25).

Hu et al. (26) first revealed the direct association between oral microbiome composition and CKD by comparing oral samples from CKD patients and healthy controls, and found that oral microbial features were correlated with inflammatory kidney biomarkers—providing preliminary evidence for the role of oral microbiota in CKD pathogenesis within the oral-urinar*y* axis framework. Although direct mechanistic evidence is still limited, the link between periodontal disease and CKD has been widely established: periodontal disease is a well-recognized nontraditional risk factor for CKD (27), and a systematic review of 5 observational studies confirmed that periodontitis is associated with an increased risk of CKD (28). After adjusting for major cardiovascular and CKD risk factors, a declining GFR (GFR < 60 mL/min/1.73 m^2^) is still associated with initial and severe chronic periodontitis (29), and CKD patients have more severe periodontitis and a higher abundance of periodontal pathogens (*Porphyromonas gingivalis*, *Tannerella forsythia*, *Treponema denticola*, *Candida albicans*) than healthy individuals (22).

Immunoglobulin A (IgA) nephropathy (IgAN), the most common primary glomerulonephritis, has a close association with oral microbiota within the oral-urinar*y* axis. Nagasawa et al. (30, 31) detected *Treponema denticola*, *Campylobacter rectus*, and *Porphyromonas gingivalis* in the tonsils of IgAN patients, and verified the pathogenicity of *Porphyromonas gingivalis* in IgAN via animal experiments, providing the first experimental evidence for the causal link between oral pathogens and IgAN. Additionally, *cnm*-positive *Streptococcus mutans* variants have been isolated from oral and tonsillar samples of IgAN patients, and animal experiments further confirmed that this strain can directly induce IgAN by mediating collagen binding and renal tissue damage (32–34).

Numerous cross-sectional studies have identified distinct oral microbial profiles in CKD and IgAN patients. Guo et al. (35) found that CKD patients had increased oral microbial diversity, with enrichment of *Streptococcus*, *Leptotrichia*, and *Actinomyces* and depletion of *Haemophilus* and *Prevotella*; however, this study has limitations such as small sample size and lack of longitudinal follow-up, and its conclusions need to be verified by large-scale prospective studies. He et al. (36) reported that *Capnocytophaga* and *SR1_genera_incertae_sedis* were enriched in IgAN patients, while 17 genera including *Rothia*, *Dialister*, and *Actinomyces* were significantly depleted, and the study further found that oral microbial dysbiosis was associated with IgAN subphenotypes, suggesting the potential of oral microbiota as a prognostic biomarker for IgAN.

Overall, the association between oral microbiota and CKD is supported by abundant observational evidence, and animal experiments have confirmed the causal role of specific oral pathogens in IgAN. However, most studies are cross-sectional with potential confounding factors, and large-scale prospective cohort studies and interventional studies are needed to confirm the causal relationship and clinical significance.

### 4.2 Oral microbiome and urolithiasis

Urolithiasis refers to the formation of calculi (stones) in any part of the urinary tract (kidneys, ureters, bladder, urethra), and kidney stones are the most common type, affecting 10%–15% of the global population (37). Patients with urolithiasis suffer from severe pain, high recurrence rate, and increased risk of renal dysfunction, hydronephrosis, and renal cell carcinoma (38).

It is known that the human microbiota are also implicated in nephrolithiasis (39), but currently, few studies have explored the association between the oral microbiome and urolithiasis—despite both being closely linked to inflammatory status and abnormal osteogenesis within the oral-urinar*y* axis. A bidirectional Mendelian randomization (MR) study by Xu et al. (11) provided the first causal evidence for the association between oral health and urolithiasis: the study used single nucleotide polymorphisms (SNPs) as instrumental variables to proxy for genetically predicted oral disease status, and found that a history of caries was negatively correlated with urolithiasis risk, while genetically predicted gingivitis was associated with a higher risk of urolithiasis. MR is a genetic epidemiological method that can minimize confounding factors and reverse causation bias, and its findings suggest a potential causal link between oral inflammatory status and urolithiasis (11).

Xu et al. (40) further compared the oral microbiome of patients with different types of kidney stones (calcium oxalate stones and uric acid stones) and healthy controls, and found that the oral microbial structure and composition were significantly different between groups: the abundance of *Porphyromonas* and *Neisseria* was higher, while *Corynebacterium*, *Leptotrichia*, and *Prevotella* was lower in stone patients; additionally, the oral microbiota richness in patients with uric acid stones was significantly decreased, with enrichment of *Neisseria* and depletion of *Veillonella* in saliva (40). This study is the first to reveal the association between oral microbiome and kidney stone subtypes, but it is a cross-sectional study with a small sample size, and the underlying metabolic mechanisms need to be further explored via multi-omics analysis.

Currently, the research on the oral microbiome and urolithiasis is limited to epidemiological and correlational evidence, and no causal or mechanistic evidence has been reported. Future studies should focus on the metabolic cross-talk between oral microbiota and kidney stone formation (e.g., oxalate metabolism, calcium regulation) and verify the potential of oral microbiome as a predictive biomarker for urolithiasis within the oral-urinar*y* axis.

### 4.3 Oral microbiome and benign prostate diseases

Benign prostate diseases mainly include prostatitis and benign prostatic hyperplasia (BPH): prostatitis is the most prevalent urological disorder in young and middle-aged men (41), while BPH is the most common urological condition in older males (42), and both are associated with chronic inflammation and impaired prostate function.

The prostate is prone to infections caused by pathogenic microorganisms present in the male reproductive system. Based on the detection of oral microbiota in genitourinary organs, the oral-genitourinar*y* axis (a key component of the oral-urinar*y* axis) has been proposed, and accumulating evidence links oral microbiota dysbiosis to benign prostate diseases (9). Multiple observational studies have confirmed that periodontal disease significantly increases the risk of BPH (43, 44), and a cross-sectional study by Wu et al. (44) further found a dose-response relationship between the severity of periodontal disease and BPH, providing stronger epidemiological evidence for the association.

Estemalik et al. (45) conducted the first direct detection of oral pathogens in prostatic secretion, and found that 70.8% of samples from patients with chronic prostatitis or BPH contained at least one oral pathogen (*Porphyromonas gingivalis*, *Prevotella intermedia*, *Treponema denticola*), and the same pathogens were detected in the subgingival plaque of the same patients, providing direct evidence for the bacterial dissemination pathway of the oral-urinar*y* axis. Additionally, *Fusobacterium nucleatum*, a key periodontal pathogen, was identified in prostates with chronic inflammation and BPH, and this pathogen has been confirmed to have strong invasiveness and the ability to induce chronic inflammation, which may be a key bridge between oral dysbiosis and prostate tissue damage (46)**.**

Clinical studies have found a significant positive correlation between prostate-specific antigen (PSA, an inflammatory marker) and periodontal parameters (clinical attachment level, probing depth, gingival index, plaque index) in patients with chronic prostatitis and periodontal disease (47, 48). Notably, an interventional study by Alwithanani et al. (49) found that periodontal treatment could significantly reduce PSA levels and improve prostate symptoms in men with high PSA and chronic periodontitis, providing the first interventional evidence for the causal link between periodontal disease and benign prostate diseases.

Overall, the association between oral microbiota and benign prostate diseases is supported by epidemiological, direct pathogen detection, and interventional evidence, and the bacterial dissemination and chronic inflammation pathways are the main proposed mechanisms. However, the number of interventional studies is small, and large-scale randomized controlled trials (RCTs) are needed to confirm the therapeutic effect of periodontal treatment on benign prostate diseases. Additionally, the role of oral microbial metabolites in prostate tissue damage needs to be further explored.

### 4.4 Oral microbiome and urologic cancers

Urologic cancers are a group of malignancies involving the prostate, bladder, kidney, penis, and testis, with prostate cancer, bladder cancer, and renal cancer being the most common types (50). They are associated with high mortality worldwide, and the role of the microbiome in tumorigenesis and progression has become a research hotspot in recent years.

Periodontitis is linked to various systemic cancers (10, 51), and the oral microbiome is considered a potential causal link between periodontitis and urologic cancers (2). However, the association between oral microbiota and urologic cancers is controversial, with conflicting findings in existing studies due to methodological heterogeneity (52). Epidemiological cohort studies have reported a positive association between periodontitis and the risk of prostate cancer (53), bladder cancer (54), and kidney cancer (55). Michaud et al. (54, 55) found that periodontal disease significantly increased the risk of renal cancer in the Health Professionals Follow-up Study (a large prospective cohort study with long-term follow-up), and the results were adjusted for multiple confounding factors (e.g., smoking, alcohol consumption, diet), with high evidence strength. In contrast, Wen et al. (56) conducted a nationwide cohort study and found no significant increase in the incidence of urologic cancers in the periodontitis cohort, and Michaud et al. (57) also found no association between periodontitis and renal cancer risk in a subgroup analysis of never smokers.

The main reasons for the conflicting findings include: (1) inconsistent diagnostic criteria for periodontal disease [most studies do not distinguish between gingivitis and periodontitis, while only periodontitis with anaerobic pathogens is associated with systemic tumors (9)]; (2) incomplete adjustment of confounding factors (e.g., smoking is a common risk factor for periodontal disease and urologic cancers); (3) differences in study populations and follow-up time (52).

To date, only a few studies have detected oral pathogens in urologic cancer tissues: Fusobacterium nucleatum has been identified in prostate cancer tissues (46), and its abundance is associated with tumor stage and lymph node metastasis, suggesting its potential role in tumor progression. However, no oral pathogens have been detected in bladder cancer or renal cancer tissues, and the mechanistic link between oral microbiota and urologic cancer remains unclear.

Overall, the association between oral microbiota and urologic cancers is still controversial, and the existing evidence is mainly limited to epidemiological studies with high heterogeneity. No causal or mechanistic evidence has been reported, and future studies should: (1) use unified diagnostic criteria for periodontal disease; (2) conduct multi-center prospective cohort studies with complete confounding factor adjustment; (3) explore the role of oral microbiota in urologic cancer via tumor tissue detection and animal experiments; (4) analyze the potential of oral microbiota as a predictive biomarker for urologic cancers—all within the framework of the oral-urinar*y* axis.

### 4.5 Oral microbiome and HSPN

Henoch-Schönlein Purpura (HSP) is the most prevalent systemic vasculitis in childhood (predominantly 2–11 years old), and HSPN is its most severe long-term complication, affecting 30%–50% of HSP patients and associated with an increased risk of chronic renal failure in adulthood (58, 59).

Infections, especially hemolytic streptococcal infections, are the main trigger of HSP (60, 61). A retrospective analysis of 2,074 pediatric HSP patients found that streptococcal infection was present in 18.9% of cases, and elimination of streptococcal infection could reduce the recurrence rate of HSP (62). Approximately 30%–50% of HSP patients develop renal injury, mostly mild but occasionally manifesting as nephrotic syndrome or renal failure (63). Schmitt et al. (64) detected IgA-bound streptococcal M protein deposits in 80% of skin biopsies and 54% of renal biopsies from HSP patients, providing direct evidence for streptococcal involvement in HSP-associated renal injury; a Japanese study further found significantly higher antistreptolysin O titers in HSP patients with renal involvement than in those with other nephropathies (65), reinforcing the streptococcus-HSPN correlation.

In the past 20 years, emerging evidence has linked oral microbiota dysbiosis to HSPN. Tahmassebi et al. (66) documented the first case of HSP after endodontic treatment, establishing an initial link between odontogenic lesions and HSP onset. Inoue et al*.* (67) conducted a clinical investigation and found a high prevalence of dental caries (70%) and apical periodontitis (53%) among HSP patients, and proposed that prompt management of odontogenic lesions may prevent HSP development—this study is the first to link oral clinical diseases to HSP, and its findings have important clinical implications for the prevention of pediatric HSP.

16S rRNA sequencing studies have identified distinct oral microbial profiles in pediatric HSP patients: Chen et al. (68) found that HSP patients had significantly higher oral microbial diversity and richness than healthy controls, with enrichment of pro-inflammatory taxa; Pang et al. (69, 70) further found that the tongue coating microbiota of HSP and HSPN patients was dominated by Firmicutes, Proteobacteria, and Bacteroidetes, and the abundance of specific taxa was associated with disease severity—suggesting the potential of tongue coating microbiota as a non-invasive biomarker for HSPN within the oral-urinar*y* axis.

Overall, the association between oral microbiota and HSPN is supported by clinical observation, microbial sequencing, and pathogen detection evidence, and streptococcal infection and oral microbial dysbiosis are the main proposed risk factors. However, most studies are cross-sectional or case reports, and large-scale prospective cohort studies are needed to confirm the causal relationship. Additionally, the mechanism by which oral microbiota modulates renal vasculitis in HSPN needs to be further explored via animal experiments and multi-omics analysis. Clinically, early management of childhood dental caries and apical periodontitis may be a potential modifiable strategy for the prevention of HSPN.

## 5 Potential mechanism of oral microbiota involved in the urinary system disease

As mentioned above, oral microbiota contribute to the pathogenesis of urinary system diseases via the oral-urinar*y* axis, and bacterial invasion, chronic inflammatory response, and oxidative stress are the three core mechanisms. Notably, the activation and manifestation of these mechanisms are disease-specific, and the same oral pathogen may exert different pathogenic effects in different urinary system diseases (9). This section elaborates on the disease-specific pathogenic mechanisms of oral microbiota in urinary system diseases, combined with the latest clinical and experimental evidence.

### 5.1 Bacteria invasion

The gingival mucosa in the periodontal region is surrounded by a dense lymphatic network with open junctions, and periodontal pockets resemble ulcers, leading to transient bacteremia during mastication, oral hygiene, or dental treatment [Figure 1B(1)] (71). Oral pathogens with strong invasiveness and virulence factors (e.g., *Porphyromonas gingivalis*, *Fusobacterium nucleatum*, *Aggregatibacter actinomycetemcomitans*) can avoid the host immune response and disseminate systemically via the bloodstream (Figure 1A) (72–74), which is the core bacterial invasion pathway of the oral-urinar*y* axis.

This mechanism is most clearly confirmed in IgAN and benign prostate diseases [Figure 1B(1)]: (1) *Porphyromonas gingivalis* and *cnm*-positive *Streptococcus mutans* can colonize the tonsils and disseminate to the kidneys, where they bind to renal collagen and induce IgA deposition and glomerular damage (30, 32–34); Komaru et al. (75) reported the first case of nephritis-associated plasmin receptor (NAPlr)-positive glomerulonephritis caused by *Aggregatibacter actinomycetemcomitans* bacteremia—providing direct clinical evidence for bacterial invasion-induced renal injury within the oral-urinar*y* axis. (2) Oral pathogens such as *Porphyromonas gingivalis* and *Fusobacterium nucleatum* can disseminate to the prostate via the bloodstream, colonize the prostate tissue, and induce chronic prostatitis and BPH (45, 46). To date, no direct bacterial invasion has been detected in CKD, urolithiasis, or urologic cancers, and this mechanism may be specific to IgAN and benign prostate diseases.

Periodontal bacterial DNA has been detected in vascular plaques (76, 77), confirming the systemic dissemination ability of oral pathogens. However, the specific tissue tropism of oral pathogens (e.g., why *Porphyromonas gingivalis* targets the kidneys and prostate) is still unclear, and may be related to the specific receptor expression on the surface of renal and prostate cells (9).

### 5.2 Inflammatory response

Inflammation is a pivotal pathogenic factor in almost all urinary system diseases, and oral microbiota can induce systemic chronic inflammation, which further promotes renal, prostate, and urinary tract tissue damage —this is the most universal mechanism of the oral-urinar*y* axis (10).

In periodontal disease, local monocytes and dendritic cells release a variety of pro-inflammatory cytokines (IL-1β, IL-6, TNF-α) (78, 79), which enter the systemic circulation and induce persistent low-grade inflammation. In CKD, elevated levels of IL-6, TNF-α, and high-sensitivity C-reactive protein (hsCRP) are associated with decreased eGFR and albuminuria, and can induce endothelin-1 and fibrotic gene expression, promoting renal fibrosis and CKD progression [Figure 1B(2)] (80–82); Liu et al. (82) found a direct correlation between periodontitis-induced inflammatory factor levels and CKD severity, providing clinical evidence for the inflammatory mechanism in CKD.

In benign prostate diseases, oral pathogen-induced systemic inflammation can exacerbate local prostate inflammation, and pro-inflammatory cytokines can promote prostate epithelial cell proliferation and BPH development [Figure 1B(2)] (44); PSA levels, a marker of prostate inflammation, are positively correlated with periodontal inflammatory parameters, and periodontal treatment can reduce both periodontal and prostate inflammation (47–49).

In urolithiasis, gingivitis-induced systemic inflammation can alter renal calcium and oxalate metabolism [Figure 1B(2)], promoting crystal nucleation and stone formation—consistent with the MR study finding that genetically predicted gingivitis is associated with a higher urolithiasis risk (11) within the oral-urinar*y* axis.

In HSPN, oral microbial dysbiosis-induced systemic inflammation can activate the immune system, induce vascular endothelial damage, and promote the development of renal vasculitis [Figure 1B(2)] (68, 69); streptococcal infection can further trigger cross-reactivity between microbial antigens and renal tissue, exacerbating IgA deposition (64, 65).

In urologic cancers, chronic inflammation induced by oral microbiota can create a tumor-promoting microenvironment, and pro-inflammatory cytokines can promote tumor cell proliferation, invasion, and metastasis [Figure 1B(2)] (51); however, this mechanism is still hypothetical and lacks direct experimental evidence (9).

Overall, the chronic inflammatory response is a universal mechanism of the oral-urinar*y* axis, and its pathogenic effect is achieved by regulating disease-specific molecular pathways. The cross-talk between oral microbiota-induced systemic inflammation and the local inflammatory microenvironment of the urinary system is the core of this mechanism, and its specific molecular network needs to be further explored via multi-omics and single-cell sequencing technologies.

### 5.3 Oxidative stress response

Oxidative stress is defined as the imbalance between reactive oxygen species (ROS) production and antioxidant defense, and is a key pathogenic factor in renal tissue damage (83). Oral microbiota can induce systemic oxidative stress, which further promotes the development of CKD and urolithiasis [Figure 1B(3)]—this mechanism has been confirmed by experimental and clinical studies (84, 85).

During periodontitis, neutrophils release a large amount of ROS to eliminate oral pathogens, and lipopolysaccharides from periodontal pathogens (*Aggregatibacter actinomycetemcomitans*, *Porphyromonas gingivalis*, *Prevotella intermedia*) can further induce ROS production by neutrophils (86), leading to systemic oxidative stress. França et al. (84) conducted the first animal experiment to confirm that experimental periodontitis can induce renal histological changes and increased oxidative stress in rats, and the renal damage is positively correlated with the severity of periodontitis; Kara et al. (85) found that melatonin, an antioxidant, can alleviate liver and kidney function damage induced by experimental periodontitis, providing direct experimental evidence for the oxidative stress mechanism in CKD.

In CKD, oxidative stress can induce renal tubular epithelial cell injury, promote renal fibrosis, and exacerbate CKD progression (81); oral microbiota-induced oxidative stress can further aggravate the oxidative damage in CKD patients, forming a vicious cycle (84).

In urolithiasis, ROS can promote renal tubular epithelial cell damage and crystal adhesion, which is a key step in kidney stone formation (39); oral microbiota-induced oxidative stress can enhance this process and increase the risk of urolithiasis (11).

To date, the role of oxidative stress in benign prostate diseases, urologic cancers, and HSPN has not been reported, and this mechanism may be specific to CKD and urolithiasis. The specific ROS sources and antioxidant defense pathways in the oral-urinar*y* axis need to be further explored via animal experiments and clinical studies to clarify their role in disease progression.

## 6 Conclusion and future perspectives

Substantial evidence indicates that oral microbiota dysbiosis is closely associated with major urinary system diseases (CKD, urolithiasis, benign prostate diseases, urologic cancers, pediatric HSPN) via the oral-urinar*y* axis, and bacterial invasion, chronic inflammatory response, and oxidative stress are the three core pathogenic mechanisms with distinct disease-specific manifestations.

Despite the significant progress in this field, several key challenges remain to be addressed:

1. Confirmation of causal relationships: Most existing studies are cross-sectional or observational, and large-scale prospective cohort studies, RCTs of periodontal treatment, and animal experiments are needed to confirm the causal link between oral microbiota and urinary system diseases.
2. Elucidation of disease-specific mechanisms: The specific molecular pathways and tissue tropism of oral microbiota in different urinary system diseases are still unclear, and multi-omics (microbiomics, transcriptomics, metabolomics) and single-cell sequencing technologies should be used to explore the underlying mechanisms.
3. Development of non-invasive biomarkers: The oral microbiome has great potential as a non-invasive biomarker for the early diagnosis and prognosis of urinary system diseases (e.g., IgAN, HSPN), and future studies should focus on identifying disease-specific microbial signatures and verifying their clinical value.
4. Exploration of microbiome-targeted interventions: Oral microbiome-targeted strategies (e.g., periodontal treatment, probiotics, fecal microbiota transplantation) have great translational potential for the prevention and treatment of urinary system diseases, and RCTs are needed to confirm their therapeutic effects.
5. Establishment of standardized research methods: The existing studies have high heterogeneity due to inconsistent oral sampling sites, microbiome detection methods, and diagnostic criteria for periodontal disease and urinary system diseases, and the establishment of standardized research methods is the key to promoting the development of this field.

In conclusion, the oral-urinar*y* axis is a novel and promising research field that provides a new perspective for understanding the pathogenesis of urinary system diseases. With the development of microbial research technologies, oral microbiome-targeted strategies are expected to become a new non-invasive approach for the prevention, diagnosis, and treatment of urinary system diseases in the near future.
